# Comprehensive framework between environment and genomic stability: the open symposium of the Japanese Environmental Mutagen Society (JEMS) in 2019

**DOI:** 10.1186/s41021-019-0132-9

**Published:** 2019-09-13

**Authors:** Katsuyoshi Horibata, Masashi Sekimoto, Kei-ichi Sugiyama

**Affiliations:** 1 Division of Genetics and Mutagenesis, National Institute of Health Sciences, 3-25-26 Tonomachi, Kawasaki-ku, Kawasaki, Kanagawa 210-9501 Japan; 2 Laboratory of Environmental Hygiene, Department of Environmental Science, School of Life and Environmental Science, Azabu University, 1-17-71 Fuchinobe, Chuo-ku, Sagamihara, 252-5201 Japan

**Keywords:** Epigenetic gene regulation, Mutagenesis, Carcinogenesis, Environmental mutagen, Genetic toxicology, Metabolomics

## Abstract

The open symposium of the Japanese Environmental Mutagen Society (JEMS), under the title of “Comprehensive framework between environment and genomic stability,” was held in the Main Conference Room of the Foundation for Promotion of Cancer Research, Tokyo, on June 8, 2019. To understand the relationship between genes and environmental mutagens, the symposium highlights the research activities in the fields of cancer, carcinogenesis and related diseases caused by genomic instabilities, including epigenetic and metabolomic alterations. The symposium was planned to help familiarize attendees with the current trends in research on genome safety. The organizers herein present a summary of the symposium.

## Background

The Open Symposium of the Japanese Environmental Mutagen Society (JEMS) is annually organized to present JEMS’ research activities in the fields of genetic toxicology and environmental mutagenesis to the public, and its proceedings are summarized in meeting reports [1–5]. The last two consecutive symposiums have been designed by and for young scientists [4, 5]. In 2019, the symposium, “Comprehensive framework between environment and genomic stability,” was designed by 6 scientists, including two JEMS members. The aim of the symposium this year was to provide a precious opportunity to consider the implications of genome safety, which can help protect against genetic diseases such as cancer. The program of the symposium highlights the research activities in the fields of cancer, carcinogenesis and related diseases caused by genomic instabilities, including epigenetic and metabolomic alterations, to understand the relationship between genes and environmental mutagens. The symposium was held in the Main Conference Room of the Foundation for Promotion of Cancer Research, Tokyo, on June 8, 2019. Through this report, the organizers present a summary of the symposium.

### Symposium program

Masamitsu Honma (President, JEMS: National Institute of Health Sciences): Inaugural speech.

Kei-ichi Sugiyama (National Institute of Health Sciences): Introduction.

Session 1 (Chairs: Kei-ichi Sugiyama and Daisuke Nakajima).

Kei-ichi Sugiyama: Epigenetic mutagen detected in environmental media.

Makoto Shibutani (Tokyo University of Agriculture and Technology): Altered epigenetic gene regulation in response to short-term carcinogen treatment and its role in carcinogenesis.

Session 2 (Chairs: Katsuyoshi Horibata and Ayumi Yamamoto).

Yuki Nagata (Tokyo Medical and Dental University): Metabolomics analyses of cerebrospinal fluid in Alzheimer’s disease and idiopathic normal pressure hydrocephalus.

Takahiro Matsumoto (Kyoto Pharmaceutical University): Isolation and structure elucidation of terpenoids as cancer prevention agents.

Session 3 (Chairs: Masashi Sekimoto and Yuji Ishii).

Kastumi Fukamachi (Nagoya City University): Animal models of carcinogenesis using RAS transgenic rats.

Rui-Sheng Wang (National Institute of Occupational Safety and Health): Study on the metabolism and toxicity of industrial chemicals using drug-metabolizing enzyme gene knockout mice.

Masashi Sekimoto (Azabu University): Concluding speech.

### Meeting report

Dr. Kei-ichi Sugiyama reported that a yeast-based bioassay, called the “FLO assay”, can detect epigenetic alterations that contribute to promoter activity of the flocculation gene *FLO1*. The endpoints of the bioassay are the degrees of flocculation and *FLO1* promoter-dependent reporter-gene activity in yeasts transformed with empty vectors or expression plasmids containing human DNA methyltransferase genes. It has already been shown that both flocculation and reporter activity can be induced by alizarin, which is considered to promote cancer. In addition, the FLO assay detected a potential epigenetic alteration mediated by inhibiting DNA methylation caused by a mycotoxin ochratoxin A, which has been classified by International Agency for Research on Cancer as a Group 2B possible human carcinogen. Taken together, these findings suggest that the FLO assay, which enables the detection of epigenetic mutagens in environmental media, has the potential to improve the assessment of the carcinogenic potential of chemicals.

Dr. Makoto Shibutani gave a talk on the identification of hypermethylated genes in the liver of rats repeatedly administered hepatocarcinogens for 28 days and their role in hepatocarcinogenesis. As the first topic, his group revealed a link between *Tmem70* downregulation and the shift from oxidative phosphorylation to glycolysis, which has long been known as the Warburg effect, for promoting cell proliferation and glycolysis through c-MYC activation. His group also suggested that the downregulation of *Ube2e2*, an ubiquitin-binding protein, stabilized the target molecules for degradation, leading to the prolonged activation of cell proliferation and delayed DNA repair for facilitating hepatocarcinogenesis. As a second topic, his group suggested that the downregulation of *Ldlrad4* promoted TGF-β signal activation as a unique mechanism for non-genotoxic hepatocarcinogenesis. These results suggest that carcinogen treatment causes the downregulation of key genes for carcinogenesis in target cells due to hypermethylation before the induction of proliferative lesions.

Dr. Yuki Nagata gave a talk on metabolomics analyses of idiopathic normal pressure hydrocephalus (iNPH) and Alzheimer’s disease (AD). iNPH is a treatable form of dementia. However, some symptoms of iNPH are similar to those of AD, making it difficult to discriminate these two diagnoses. Therefore, to identify new biomarkers in the cerebrospinal fluid for these two diseases, Dr. Nagata’s group performed metabolomics analyses and identified four metabolites able to distinguish iNPH and AD. These metabolites were deeply involved in the biological pathways associated with AD. Metabolic disorder is a cause of AD, so it would be reasonable to use metabolic biomarkers as early markers of AD. However, there is no curative treatment for AD, so it is important to prevent AD. Recently, genetic polymorphisms have been revealed to be involved in the risk of various diseases. Genomic analyses will become a useful tool for screening people with an increased risk of AD. In the future, such people will be trained on appropriate methods for preventing AD, which will help reduce the rate of dementia.

Dr. Takahiro Matsumoto presented the isolation and structure elucidation of cancer prevention agents from edible plants, such as *Citrus limon* and *Petasites japonicus*. Through his isolation efforts, he elucidated the chemical structures of more than 20 new compounds. The antimutagenic effects of the isolated compounds were evaluated using the Ames test and in vivo micronucleus test. In addition, some isolated compounds showed cancer stem cell (CSC) growth inhibitory effects. CSCs are resistant to many anticancer drugs and play an important role in metastasis. This research project will provide important information for cancer prevention.

Dr. Kastumi Fukamachi presented RAS transgenic rats as short-term carcinogenesis models. These rats are available from CLEA Japan (Tokyo, Japan). His group has generated human c-Ha-ras proto-oncogene transgenic (Hras128) rats that are highly sensitive to mammary carcinogens. Various carcinogens, not necessarily just those normally targeting the breast, were found to induce mammary carcinomas in Hras128 rats. These results support the possible application of these rats as an animal model for the short-term evaluation of the carcinogenicity of chemical compounds. Furthermore, his group established a rat model of human pancreatic ductal adenocarcinoma, in which the expression of the human mutated Kras oncogene and induction of pancreas cancer were regulated by the Cre/lox system. Neoplastic lesions exhibit morphological and biological similarities to those observed in human pancreas lesions. Rat models of pancreatic cancer will be useful for screening candidate chemotherapeutic agents or diagnosis markers of human cancer.

Dr. Rui-Sheng Wang presented the results of his studies of the metabolic pathways related to the genotoxicity of industrial chemicals using drug-metabolizing enzyme gene-knockout mice. With CYP2E1-knockout mice, his group found that this enzyme is responsible for the primary metabolism of 1,2-dichloropropane, a solvent suspected of causing cases of bile duct cancer in an offset printing factory. CYP2E1 is essential for the induction of liver injuries and DNA damage after 1,2-dichloropropane administration, and methylglyoxal, a genotoxic substance, was identified as an intermediate metabolite of the solvent. High concentrations of this metabolite in the bile suggest its possible contribution to the occurrence of cancer among workers. In studies on styrene with ALDH2 gene knockout mice, severe DNA damages was detected in the liver, but the concentration of metabolites in the urine was lower than in wild-type mice. Studies using these knockout mice models have provided useful information on the mechanisms underlying carcinogenesis due to industrial chemical exposure.

There were approximately 100 participants at the symposium. As the organizers, we would like to thank to everyone who attended this symposium.

## Abbreviations

AD: Alzheimer’s disease
CSC: Cancer stem cell
Hras128: Human c-Ha-ras proto-oncogene transgenic
iNPH: Idiopathic normal pressure hydrocephalus
JEMS: Japanese Environmental Mutagen Society
